# Sociodemographic and Clinical Characteristics of Centenarians in Mexico City

**DOI:** 10.1155/2017/7195801

**Published:** 2017-08-17

**Authors:** Valdés-Corchado Pedro, Ruiz-Hernández Arturo, Pérez-Moreno Alejandro, Rosas-Carrasco Oscar

**Affiliations:** ^1^Institute for the Care of Older Adults in the Federal District, Mexico City, Mexico; ^2^National Geriatrics Institute, National Institutes of Health, Periférico Sur No. 2767, Col. San Jerónimo Lídice, Del. Magdalena Contreras, 10200 Mexico City, Mexico

## Abstract

**Background:**

There is little evidence about the demography and health status of adults aged 100 years and over in Latin America and there are no studies in Mexico.

**Objectives:**

To describe the demographic characteristics and health status of centenarians residing in Mexico City.

**Methods:**

This is a cross-sectional study using a population base of 393 community-dwelling centenarians in Mexico City. A comprehensive geriatric assessment was performed, including demographic information and health status.

**Results:**

The mean age of centenarians was 101.82 ± 2.02 years, of whom 44 (9.1%) were semisupercentenarians (105–109 years old) and 5 (0.2%) were supercentenarians (≥110 years old). The female/male ratio was 3.2 : 1. Twelve (4.5%) reside in nursing homes. Women versus men have unfavorable conditions given their criteria: being without a partner, dependence in 1 or more basic activities, dependence in 1 or more instrumental activities, hypertension, cancer, and Parkinson's disease. Nevertheless, as compared to other populations, Mexican centenarians report having good self-perception of health (78.9%), polypharmacy (17.8%), low rate of pain (11.4%), diabetes (4.8%), and dyslipidemia (1.8%).

**Conclusions:**

This is the first study in Latin America that describes the social and clinical characteristics of centenarians in Mexico City. This population has a high percentage of malnutrition and osteoarthrosis, a high self-perception of health, low frequency of diabetes, dyslipidemia, cardiovascular disease, and a high frequency of “escapers” (24%).

## 1. Introduction

The Mexican population, aged 60 years and older, comprises 10,055,379 people, or 9.06% of the total country's population (6.3% of those aged 65 years or older) [1]. Life expectancy has risen: 77.4 years for women and 71.7 years for men [1]. Mexico City is the district with the highest proportion (1,003,648 people, 11.3%) of older adults in the country, according to the most recent figures of the 2010 Population and Housing Census in Mexico [1].

Given that life expectancy has risen, cases of exceptional longevity are increasingly frequent. Centenarians constitute the best model of prolonged aging; they are survivors, having overcome the diseases that affect people in different stages of life, thanks to the many mechanisms that have been developed to allow people to overcome these obstacles at different levels: medical, psychological, and social [2].

Different studies have reported on the clinical and sociodemographic characteristics of centenarians in European [3–5] and Asian [6–8] countries as well as the United States of America [9, 10], among others; however, very few studies in Latin America have been published [11, 12].

These studies have stated the social, clinical, and psychological characteristics of centenarians in each population studied. Learning about the status of centenarians in less industrialized nations, such as Mexico, is exceedingly interesting, for these are individuals who have faced diverse factors over the course of their life that would likely impact their health. Describing these factors and characteristics is the primary purpose of this study.

## 2. Materials and Methods

### 2.1. Study Population

This is a cross-sectional study where most of the +100-year-old residents of Mexico City were assessed. Selection was based on the food pension registry given by the Government of Mexico City to all older adults, 68 years and older, and based on residency in this city by the Institute for the Care of Older Adults in the Federal District (IAAMDF), through which 781 centenarian adults were listed in the year 2010. According to the Population and Housing Census [13], it is estimated that this registry lists 80% of the population that is 68 years of age and older. Of the 781 centenarians registered through the year 2010, 393 were included in the study once they gave positive proof of age; these 393 subjects come from all 16 districts that comprise Mexico City.

All centenarians and/or caregivers were interviewed and examined in their homes by general practitioners who had received prior standardized training by geriatricians. They applied a comprehensive geriatric assessment that included sociodemographic characteristics and geriatric scales. All the centenarians were included and interviewed personally except when the Mini-Mental State Examination had a score of ≤10 points or when there was a severe visual or hearing impairment, in which case the interview was answered by caregivers. Centenarians and caregivers were informed of the study and signed a letter of consent.

### 2.2. Measurements

#### 2.2.1. Age

Age was validated through the voter's ID card. In Mexico, to obtain this ID, it is necessary to show the birth certificate. To strengthen the age validation, we exclude the elderly women who had biological children aged 45 years or less or older men with biological children aged 55 years or less. We excluded them in both cases if the difference in age between the centenarians and the couple was not more than 30 years.

Other sociodemographic variables were also collected: gender (*man, woman*), marital status (*no partner*: single, widow (er), divorcé (e), separated;* with partner*: married, common law partner); schooling was determined in years starting with basic education (*no schooling*: 0 years of schooling;* some schooling*: 1 to 6 years and ≥7 years); the relationship of the person who lives with the centenarian was also noted.

#### 2.2.2. Mini-Mental State Examination (MMSE)

This was used to assess cognitive function. This scale has been validated and used in our population [14], and its scores range from 0 to 30. Cognitive impairment was defined by score ≤23 if schooling was 5 years and more, ≤19 if schooling was between 1 and 4 years, and ≤16 if schooling was less than 1 year [14].

#### 2.2.3. Katz's Scale

This 6-item measure was used to evaluate the patient's basic activities of daily living (ADLs). The total score was dichotomized as follows: 0 when all activities were performed partially or totally independently and 1 if at least one item activity is not performed [15, 16].

#### 2.2.4. Lawton's Scale

This 8-item measure was used to evaluate the patient's instrumental activities of daily living (IADL) for women and a 5-item measure was used for men. The total score was dichotomized as follows: 0 = when all the activities were performed partially or totally independently and 1 = if at least one item activity is not performed [17].

#### 2.2.5. Self-Perception of Health

This concept was explored by asking, “What do you consider your general health status to be at present?” Originally, there were five categories to the answer, which to facilitate analysis were grouped into two, namely,* good health status *(excellent, very good, and good) and* poor health status *(poor and very poor). This question has been used previously in different studies [18, 19].

#### 2.2.6. Geriatric Depression Scale (GDS)

This 15-item measure was used to assess depression in centenarians and it has been validated in our population; scores range from 0 to 15 and we used a cutoff of 6 or more for depression according to previous reports in older adults in Mexico [20].

#### 2.2.7. Mini Nutritional Assessment (MNA)

The MNA comprises 18 items. The MNA has already been validated in Spanish [21]. Using the total score, a variable with three categories was created: (1) good nutrition (≥24 points), (2) risk of malnutrition (from 17.5 to 23.5 points), and (3) malnutrition (≤17 points).

#### 2.2.8. Diseases

The centenarian and/or caregiver were directly asked if they had ever been diagnosed by a physician with any of the following: osteoarthrosis, hypertension, heart disease (ischemic, heart failure), actual pain, chronic obstructive pulmonary disease, acute respiratory disease (pneumonia, influenza), cerebrovascular disease (hemorrhagic, ischemic), osteoporosis, type 2 diabetes mellitus, Parkinson's disease, any type of cancer, dyslipidemia, benign prostatic hyperplasia, hypothyroidism, chronic kidney disease, actually pain, and hepatic disease. Results were recorded as either presence or absence of the disease.

#### 2.2.9. Polypharmacy and Medication

This information was collected through a checklist questionnaire. For the purposes of this study, the total number of different medications ingested on a daily basis was analyzed. Polypharmacy was defined as the ingestion of ≥5 medications, according to the predictive validity for mortality in a previous study [22].

### 2.3. Data Analysis

The mean and standard deviation for continuous variables were determined, as well as the frequency and percentages for nominal variables. The Chi-square test was used to analyze the differences in percentages. When the frequency of a cell of information was lower than 5, Fisher's exact test was used. Though the continuous variables did not show a normal trend through the Shapiro-Wilk test and graphics, some variables are reported in this paper through mean and standard deviation to facilitate their interpretation and comparison with other populations.

### 2.4. Ethics

This study is considered as a low risk study according to the General Health Law in Mexico. This study was recorded at the Institute for the Care of Older People in Mexico City and it was conducted according to the ethical and scientific standards in accordance with the provisions of the International Ethical Guidelines for Research on Human Beings of the World Health Organization. All participants signed the informed consent form.

## 3. Results

Of the 781 centenarians registered, 70 (8.9%) were deceased and 214 (27.4%) were not present at their place of residence or declined to participate in the study, whereas the ages of 104 of them (13.3%) could not be validated. The total number of centenarians included in the study was 393 (50.3% of the centenarians registered). The overall mean age was 101.82 ± 2.02 (range: 100 to 112 years); in Table 1, we can observe that there are no differences between men and women for every year of age. The mean schooling was 2.79 ± 3.60. Upon being grouped into categories, differences were not found between men and women (Table 1). As concerns marital status, men state that they have a partner more frequently than women do (Table 1).

Regarding relationship with the person with whom they live, men stating that they live alone comprised 3.1% whereas women comprised 4%. Interestingly, only 5.3% of men and 4.3% of women were living in a nursing home without significant differences. Although the differences were nonsignificant, men were living more frequently (76.6%) with their partner or children than women (66.5%). More women were living with other relatives (20%) than men (11.7%) (Table 1).

Regarding cognitive status, the overall MMSE mean was 19.49 ± 6.43 (women: 19.0 ± 6.4; men: 20.9 ± 6.2); using the stated cutoff points for the general population in Mexico, only 118 (53.1%) had normal cognitive function. No differences were found between men and women (Table 2).

Table 2 indicates that there is a high functional dependency in the total population for IADL and ADL, and there is greater dependency significant in women (Table 2).

Self-perception of health was good in men and women, without differences (Table 2). Total points on the GDS scale added up to 4.4 ± 2.87, with no differences found between the sexes. Total average MNA was 19.09 ± 4.75 (Table 2) without differences found between the sexes.

Twenty-five percent of participants reported not having any disease at all (Table 3). The mean of the number of diseases was 1.4 ± 1.2. The most frequent disease noted turned out to be osteoarthrosis, reaching 36.3%. The diseases that displayed greater differences between men and women were hypertension, Parkinson's disease, and cancer, all with a higher proportion in women.

As concerns the number of medications used, the mean was 2.39 ± 2.36; polypharmacy was recorded for nearly 17.8% of centenarians of both sexes without differences (Table 3).

## 4. Discussion

In this study, the data of all centenarians living in Mexico City in the year 2010 were analyzed. This is the first population based study in Latin America on centenarians. As concerns age, men and women were evenly distributed, ranging from 100 to 112 years. This number (*n* = 393) of centenarians which corresponds to the ratio of 0.88 per 20,000 inhabitants or 4.4 per 100,000 inhabitants is similar to Greek centenarians [4].

We found only 36 (9.1%) semisupercentenarians (105–109 years old) and 1 (0.2%) supercentenarian (≥110 years old); regarding this point, Young et al. [23] reported approximately one living supercentenarian per five million people in developed countries and far fewer in less developed countries. These data are similar to our study because in 2010 we found fewer supercentenarians; 0.56 supercentenarians per five million people (8,851,080 inhabitants in Mexico City reported by National Census 2010) [13]. These differences could be caused by the type of age validation, sampling method, distribution, and development of the population.

According to the ratio of male/female centenarians in this study, 1 : 3.2 was similar to Greece (1 : 3.3) [4] and Denmark (1 : 3.6) [24]. Regarding schooling, we found the mean to be below 3 years; this is a very low figure compared to other populations with more economic development [25]; it was higher for men than for women (without significant differences); this information is in keeping with the social and economic situation that prevailed in Mexico 100 years ago, during a period of war and after war (the Mexican Revolution of 1910); this was a highly unfavorable environment, particularly for women, as concerns attending school. Higher schooling levels for men than women are also noted in other populations [4].

Centenarians live, primarily, with relatives; very few live alone or in nursing homes (Table 1). This fact differs from that reported in other countries where there are a higher percentage of centenarians living in nursing homes (between 50% and 60%) [26, 27]. This low percentage is similar to that reported in the Greek [4, 28].

Living at home can be readily explained given that in Mexico the predominant type of family is the nuclear and extended family, representing 88% [29].

Women were more dependent in ADLs and IADLs than men. While more women attain this age, overall conditions are less favorable for women than for men. For this and other reasons, some authors have considered centenarian men to be the ideal longevity model [2].

Regarding the cognitive status of centenarians with the cutoff points in the Mexican population [14], only 53.1% had normal cognitive function; this finding was higher than those reported in other populations (19.7% in Tokyo [27] and 22.5% in Georgia [25]) and lower than in Australia (66%) [26]. This high frequency of reported cognitive impairment may be due to the use of the MMSE, which is affected by low schooling and an older age; there is no screening scales validity in centenarians in Mexico [14]. Another possible explanation for the high prevalence found of cognitive impairment is that depression was of high frequency (29%) and depression is an associated factor and a cause of cognitive impairment; however, this study is limited by the use of screening scales, which do not allow further study. Future studies will corroborate, through clinical evaluation, neuroimaging and biomarkers, the causes of this cognitive impairment.

The frequency of depression symptoms (29%) was similar to the figures found in Mexican studies (28%) where the mean was 80 years of age [20]. This fact could be explained by the generally reported good health and social characteristics (living with another person and home, low comorbidity, good self-perception, etc.) [30]. A salient fact about Mexican centenarians is that 78% of men and women perceive themselves to be in good health, compared to centenarians in Greece, where good health perception (excellent, very good, and good) stood at only 27% [28]. This good self-perception in Mexican centenarians may be the result of the low frequency of multimorbidity. In this same vein, in other qualitative reports, centenarians declare that they “feel fine.” They report that they feel good, regardless of their health status [31]. Studies on the factors associated with a good self-perception of health status in Mexican centenarians do not exist and could yield valuable results.

Malnutrition is one of the main problems present in older Mexican adults, as has been demonstrated in earlier national surveys. This situation is more noticeable in women [32] and is in keeping with other populations where women lose more weight than men [4].

The number of diseases found in this study by self-reports is similar for men and women; 24% of centenarians might be considered “escapers” (defined as centenarians presenting no disease upon turning 100 years old). This percentage is similar to that found in another study [33].

As pertains to disease, osteoarthrosis holds the first place in this population. Although there is a lot not yet known regarding the pathophysiology of the disease and its link to age, earlier reports show its high frequency in this age group, and different mechanisms associated with the extended lifespan of chondrocytes are a key to future studies [34, 35].

Hypertension is more prevalent in women (37.4%); this percentage was lower than that reported previously in a Mexican national survey (41.3%) [32]. Cardiovascular disease is present in approximately 15%, a figure much lower than that reported in earlier studies [36]; variability in measuring affects the result and self-reporting favors underdiagnosis [36]. However, the delay of these illnesses' presentation is in accord with the age reported.

Actual pain affects around 11% of the centenarians; this figure is lower than that reported previously for Mexican adults 50 years of age and older [37]. The main cause of pain in older adults is osteoarticular disease [38, 39].

As concerns other diseases, diabetes (4.8%) and dyslipidemia (1.5%) were noted to have a very low frequency rate in centenarians. In the case of diabetes, a percentage of 7.34% has been self-reported in the Mexican general population and 21% has been reported in people above 70 years of age [40, 41], not taking into account the underdiagnosis, which could be another reason for their survival. Dyslipidemia self-reported by centenarians was lower than in the Mexican general population; other authors have previously reported in Mexico that only 8% knew the diagnosis of dyslipidemia; this point limits comparison with other studies [42].

Parkinson's disease (PD) was reported only by 2.3% of women; although this survey was conducted by a general practitioner, misclassification of PD remains possible, although this low frequency of PD coincides with previous studies: PD gradually increases to 90 years and then declines precipitously [43, 44].

Polypharmacy (mean of medications) used by centenarians is lower than in other populations [45, 46]. One possible explanation for this is that multimorbidity and depression are low in our population; these are factors linked to higher prescription rates.

There are some limitations to consider: this is a cross-sectional study; it includes only descriptive data, and given the great wealth and diversity of the various geographic areas of Mexico, these results are restricted to the urban and semiurban population of major cities like Mexico City. The response rate of the study was not high (51%), which could have led to a response bias. It is possible that those who did not participate in the study may have some different characteristics than those who participated. The method for age validation used in this study through the census, the voter's ID card, and birth certificate was different from other studies; future studies in Latin America should take into account extensive age validation methodologies [47].

However, this is the first descriptive study in Mexico and Latin America about some clinical and social characteristics of centenarians. This group had a low frequency of depression, diabetes, dyslipidemia, and cardiovascular disease and a high frequency of “escapers.” Differences between men and women were found as previously reported in the world (female centenarians present a poorer health status in areas such as cognitive impairment and ADL dependence as compared to men, though their self-perception remains high).

## Figures and Tables

**Table 1:** Sociodemographic characteristics in Mexican centenarians broken down by gender.

Variable	Total *n* (%) 393	Women *n* (%) 299 (76.0)	Men *n* (%) 94 (23.9)	*p*
Age (years)				0.54
100	125 (31.8)	88 (29.4)	37 (39.3)	
101	90 (22.9)	69 (23.0)	21 (22.3)	
102	64 (16.2)	52 (17.3)	12 (12.7)	
103	48 (12.2)	39 (13.0)	9 (9.5)	
104	29 (7.3)	23 (7.6)	6 (6.3)	
≥105	37 (9.4)	28 (9.3)	9 (9.5)	
Schooling (years)				0.25
0 years	182 (46.3)	145 (48.4)	37 (39.3)	
1–6 years	170 (43.2)	125 (41.8)	45 (47.8)	
≥7 years	41 (10.4)	29 (9.7)	12 (12.7)	
Marital status				<0.001
With partner	22 (5.59)	6 (2.0)	16 (17.0)	
Without partner	371 (94)	293 (97.9)	78 (82.9)	
Living with				0.33
Partner or children	371 (94.4)	199 (66.5)	72 (76.6)	
Other relatives (grandchildren, brothers, etc.)	271 (69.0)	60 (20.0)	11 (11.7)	
Nonrelatives (neighbors, friends, etc.)^&^	18 (4.6)	15 (5.0)	3 (3.1)	
Alone^&^	15 (3.8)	12 (4.0)	3 (3.1)	
Nursing home^&^	18 (4.6)	13 (4.3)	5 (5.3)	

Differences in percentages were analyzed through the Chi^2^ test except for those marked by “&” which were analyzed through Fisher's exact test.

**Table 2:** Frequency of health characteristics in Mexican centenarians by gender.

Variable	Total *n* (%) 393	Women *n* (%) 299 (76.0)	Men *n* (%) 94 (23.9)	*p*
MMSE cognitive status				0.23
Normal cognitive status	209 (53.1)	154 (51.5)	55 (58.5)	
Cognitive impairment	184 (46.8)	145 (48.4)	39 (41.4)	
Dependence in basic activities (Katz index)				<0.01
No dependence	115 (29.2)	78 (26.0)	37 (39.3)	
Dependence in 1 or more activities	278 (70.7)	221 (73.7)	57 (60.3)	
Dependence in instrumental activities (Lawton scale)				0.007
No dependence^&^	10 (2.5)	4 (1.3)	6 (6.3)	
Dependence in 1 or more activities	383 (97.4)	295 (98.6)	88 (93.6)	
Depression by GDS				0.21
No depression, ≤5 points	277 (70.4)	206 (68.9)	71 (75.5)	
Depression, ≥6 points	116 (29.6)	93 (31.1)	23 (24.4)	
Self-perception of health status				0.53
Good	310 (78.9)	238 (79.6)	72 (76.6)	
Poor	83 (21.1)	61 (20.4)	22 (23.4)	
Nutritional status by MNA				0.33
No malnutrition	68 (17.3)	49 (16.3)	19 (20.2)	
Risk of malnutrition	206 (52.4)	154 (5.5)	52 (55.3)	
Malnutrition	119 (30.2)	96 (32.1)	23 (24.2)	
Polypharmacy				0.81
Yes (≥5 medications)	70 (17.8)	54 (18.0)	16 (17.0)	

MMSE: Mini-Mental State Examination; GDS: Geriatric Depression Scale; MNA: Mini Nutritional Assessment. Differences in percentages were analyzed through the Chi^2^ test except for those marked by “&” which were analyzed through Fisher's exact test.

**Table 3:** Frequency of the main illnesses and multimorbidity in Mexican centenarians by gender.

Variable	Total *n* (%) 393	Women *n* (%) 299 (76.0)	Men *n* (%) 94 (23.9)	*p*
Multimorbidity				0.35
0 diseases	98 (24.9)	79 (26.3)	19 (22.2)	
1 disease	133 (33.8)	102 (34.0)	31 (33.9)	
≥2 diseases	162 (41.2)	118 (39.6)	44 (41.4)	
Osteoarthrosis (yes)	143 (36.3)	104 (34.7)	39 (39.4)	0.37
Hypertension (yes)	136 (34.6)	112 (37.4)	24 (25.5)	0.01
Benign prostatic hyperplasia (yes)	28 (29.7)	—	28 (29.7)	—
Cardiovascular disease (yes)	59 (15.0)	43 (14.3)	16 (17.0)	0.53
Pain (yes)	45 (11.4)	35 (11.7)	10 (10.6)	0.77
COPD (yes)	32 (8.1)	21 (7.0)	11 (11.7)	0.14
Acute respiratory disease (yes)	31 (7.8)	23 (7.6)	8 (10.6)	0.36
Cerebrovascular disease (yes)	22 (5.5)	14 (4.6)	8 (8.5)	0.15
Osteoporosis	18 (4.5)	16 (5.3)	2 (2.1)	0.19
Type 2 diabetes (yes)	19 (4.8)	12 (4.0)	7 (7.4)	0.17
Parkinson's disease (yes)	9 (3.0)	9 (3.0)	0 (0)	—
Cancer (yes)^&^	17 (4.3)	16 (5.3)	1 (0.9)	0.04^&^
Dyslipidemia (yes)^&^	7 (1.8)	5 (1.6)	2 (1.0)	0.44^&^
Chronic kidney disease (yes)^&^	6 (1.5)	5 (1.3)	1 (2,1)	0.55^&^
Hypothyroidism (yes)^&^	4 (1.0)	4 (1.3)	1 (1.0)	0.65^&^
Hepatic disease (yes)^&^	2 (0.6)	2 (0.6)	0	.

Differences in percentages were analyzed through the Chi^2^ test except for those marked by “&” which were analyzed through Fisher's exact test.
